# Using the Footfall Sound of Dairy Cows for Detecting Claw Lesions

**DOI:** 10.3390/ani9030078

**Published:** 2019-03-01

**Authors:** Nina Volkmann, Boris Kulig, Nicole Kemper

**Affiliations:** 1 Institute for Animal Hygiene, Animal Welfare and Animal Behavior, University of Veterinary Medicine Hannover, Foundation, 30173 Hannover, Germany; nicole.kemper@tiho-hannover.de; 2 Section of Agricultural and Biosystems Engineering, University of Kassel, 37213 Witzenhausen, Germany; bkulig@uni-kassel.de

**Keywords:** lameness detection, dairy cattle, acoustic analysis, footfall sound

## Abstract

**Simple Summary:**

Claw lesions affect the health and well-being of dairy cows and are the third most frequent reason for culling. The most common method to identify cows affected by claw lesions is the assessment of locomotion. However, this requires apart from a trained observer a lot of time. And time is an important limiting factor especially with increasing herd sizes. Thus, regarding the detection of claw lesions, a sensor-assisted, automated system to be implemented on farms should be developed. This system analyzes the footfall sound of cows to distinguish lame from non-lame animals. Using a runway with sensors for recording the footfall sound, we found that cows with non-infectious diseases such as sole ulcers showed a less forceful gait pattern than healthy ones. It is known that particular cows with non-infectious diseases have a greater sensitivity to pain. Therefore the established system is capable of selecting cows that take more cautious steps. This character of abnormal gait pattern is in turn a sign of lameness. Therefore, the automated system as developed in this study is a promising tool for detecting lameness in dairy cows on farms.

**Abstract:**

An important factor for animal welfare in cattle farming is the detection of lameness. The presented study is part of a project aiming to develop a system that is capable of an automated diagnosis of claw lesions by analyzing the footfall sound. Data were generated from cows walking along a measurement zone where piezoelectric sensors recorded their footfall sounds. Locomotion of the animals was scored and they were graded according to a three-scale scoring system (LS1 = non-lame; LS2 = uneven gait; LS3 = lame). Subsequently, the cows were examined by a hoof trimmer. The walking speed across the test track was significantly higher in cows with LS1 compared to those with LS2 and LS3 and thus, they were showing a smoother gait pattern. The standard deviation of volume (SDV) in the recorded footfall sound signal was considered as a factor for the force of a cow’s footsteps. Cows with non-infectious claw lesions showed lower SDV than healthy cows and those with infectious claw diseases. This outcome confirmed the hypothesis that the evaluated cows affected by non-infectious claw lesions have a greater sensitivity to pain and demonstrate a less forceful gait pattern. These first results clearly show the potential of using footfall sound analysis for detecting claw lesions.

## 1. Introduction

Lameness is a widespread health problem in dairy production. The first step in reducing lameness is to diagnose it [1]. Therefore early detection is an important factor for animal welfare. Most commonly, lameness is caused by diseases of the claws and limbs [2]. In turn, these diseases cause pain to the animals and that leads to a change in their natural behavior and their normal gait to reduce discomfort [3,4,5]. As the cows try to relieve the affected and painful limb they change the weight distribution by shifting their weight to a healthy leg. Furthermore, they take shorter and more careful steps. However, before the stimulus becomes severe, cows tend to mask the signs of pain since they are a prey species [6]. Thus, lameness detection by visual observation is complicated, in particular, with the ever increasing size of dairy farms and the reduced time available for observing the cows [7]. For this reason, there is growing interest by dairy farmers in supporting automated methods to assist them with lameness detection. Previous studies used the changes in the normal movement as mentioned above to develop various tools with the aim of improving the assessment of lameness. Kujala et al. (2008) analyzed measurements of force sensors which recorded the weight on each leg while the cows were standing in the milking robot [8]. Their system was particularly able to detect severely lame cows affected by non-infectious diseases such as sole ulcers and white line disease. As a major drawback they concluded that these force sensors need to be checked regularly and frequently need to be repaired. Likewise, Chapinal et al. (2010) concluded that measures of weight shifting between legs have the potential to detect lameness [9]. They also used a weighing platform while the cows were standing. Maertens et al. (2011) analyzed data from cows while walking over a force-sensitive mattress and presented a kinematic gait analysis [10]. They suggested further research concerning variables of asymmetry and speed for detecting lameness was required. Other approaches for gait analysis include computer vision concepts which use information from video recordings and evaluate movement pattern such as the distance of the hind hoof from the fore hoof position [11], the arching of a cow’s back [12] or the touch and release of the fetlock joint [13]. Recently, Haladjian et al. (2018) provided a new motion-based lameness detection approach using a single motion sensor which is attached to a hind limb [14]. This wearable sensor learns the normal gait pattern of each individual cow and takes it into consideration to indicate an anomaly of movement. Nevertheless, for a practical application the system has to be validated for a longer period and on farm. Furthermore, the wearable system of Haladjian et al. (2018), which should be a low-cost-system, has to be improved regarding energy consumption and battery duration [14]. To our knowledge, currently no practical systems for an early and automated diagnosis of claw lesions exist. Therefore, the aim of our study was to provide first insights into implementing a reasonably priced system which is capable of distinguishing healthy animals from those affected by claw lesions by analyzing their footfall sound on farm. Walking on a solid surface as can be found in most cattle housings produces sound signals. It was hypothesized that this footfall sound varies in various parameters according to the gait of the animals. Therefore, in this study as selected parameters the walking speed as well as differences in the maximum volume of the footfall sound representing the weight loaded on each leg were implemented and evaluated in detail in order to detect lame cows.

## 2. Materials and Methods

### 2.1. Data Collection

**Figure 1:**
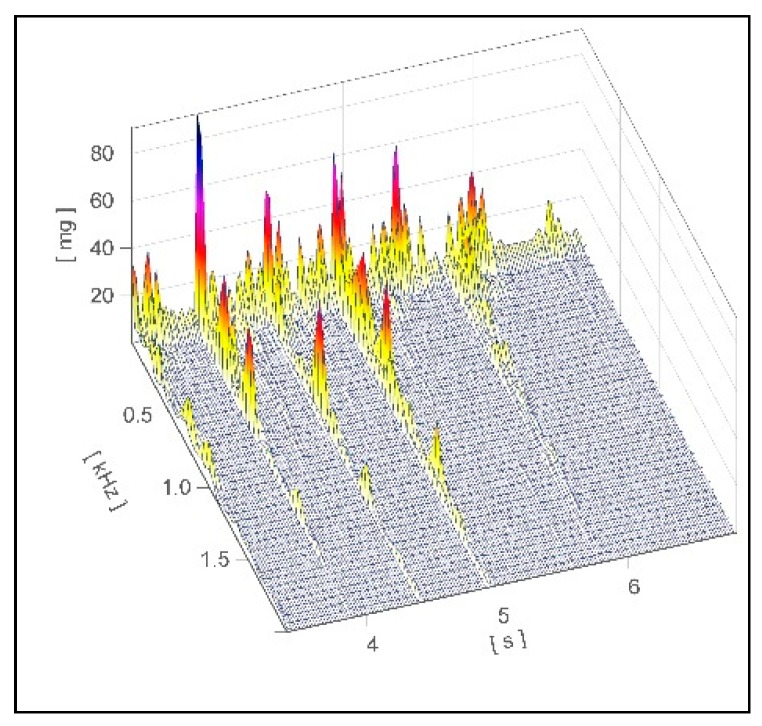
Image of a raw footfall sound file recorded on the test track using the measuring device IMC^®^ CS-3008-N.

For this study no ethical approval had to be obtained because it did not involve a prospective evaluation or laboratory animals and only non-invasive procedures were applied. The study was conducted in a free-stall barn where the lactating cows (German Holstein) were housed in two groups in two sections. The stall of group 1 consisted of slatted flooring and milking was performed by a milking robot (n = 60). Group 2 was housed in a deep straw barn and was milked twice a day in a milking parlor (n = 84). Further information about cows’ parity was included in the evaluation from herd management software (BOVI-CONCEPT, Metzner-Software, Weichs, Germany). The experimental set-up was placed outside the stable with a 22 m long test track which was partly covered with slatted floor elements (total length: 15.5 m; width: 0.8 m) separately laid (Supplementary materials: Figures S1 and S2). In the middle of the three slatted floor elements (length: 3.1 m; wide: 0.8 m) eight piezoelectric sensors (ICP accelerometer; PCB Group Inc., New York, USA) were attached and the signals of the footfall sound of the cows were recorded with a measuring device of the company IMC^®^ (imc CS-3008-N, imc Test & Measurement GmbH, Berlin, Germany) with a frequency of 50 kHz. Furthermore, rubber buffers were placed under each corner of the slatted floor elements to enable a free oscillation of the single element. The raw footfall sound files (Figure 1) were edited using the IMC^®^ Famos Signal Analysis 7.0 software (imc Test & Measurement GmbH; Berlin; Germany) and passed through a filtering process. The main focus of this filtering process was the detection of the steps and the differentiation of step events from the background noise of the entire experimental set-up. A sound pressure threshold and the direction of the slope of the amplitude were applied as filter criteria. 

**Figure 2:**
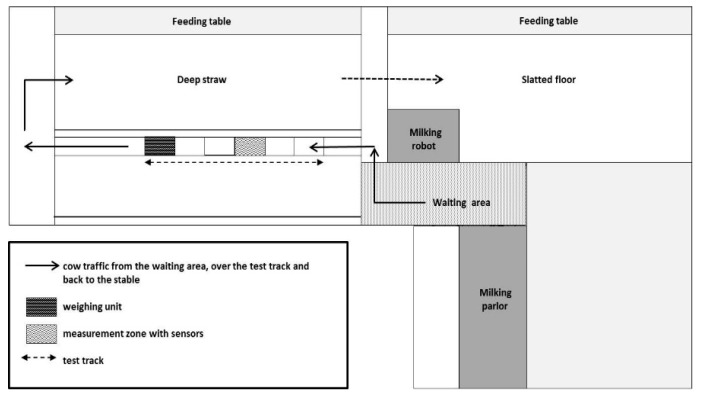
Floor plan of the two housing groups inside the stable, the milking parlor, the milking robot and the test track outside the barn with the scale and the measurement zone. The arrows show the walking direction from the waiting area over the measurement zone and back to the housing group.

At the end of the described test track the weight of each cow was measured with a weighing platform (ID5000, Tru-Test Limited, Alberta, Canada). To habituate the cows to this new gangway they had to pass the test track at least twice before measuring began. Cows from group 1 (deep straw) had to walk from the waiting area along the test track outside and back to their barn. Group 2 animals (slatted floor) walked from the waiting area over the test track and then through the deep straw to get back to their barn. During the test run of group 2 the animals from group 1 were separated in the feeding alley. The floor plan with the cow traffic is shown in Figure 2.

After the trial runs for training all lactating cows walked consecutively along the test track to record their footfall sound signals. They were filmed laterally by video (Camcorder GC-PX100BE, JVC KENWOOD Corporation, Kanagawa, Japan). Simultaneously to these recordings the animals were rated by a trained observer with a previously validated three-stage locomotion scoring system [15]. Animals with a confident walk and a perfect gait pattern were rated with score 1 (LS1). Cows with a slightly asymmetric gait and unequal weight-bearing were assessed with score 2 (LS2). Clearly lame animals on one (or more) limbs were given score 3 (LS3). Three days after recording and locomotion scoring the hooves were examined by a professional hoof trimmer in compliance with functional hoof trimming. All diagnostic findings concerning claw disorders were directly recorded in accordance with the keycode of the German Agricultural Society (DLG) [16]. Therefore, on the basis of the diagnosed type of claw lesions from all four legs the animals were grouped according to a three-level system as follows:

Group 0: cows without any claw disease;

Group 1: cows affected by non-infectious claw lesions;

Group 2: cows affected by infectious claw lesions.

If the cows were affected by both infectious and non-infectious claw diseases, they were rated with regard to the severity of the disease and, subsequently, grouped concerning the claw lesion with the higher severity. 

### 2.2. Statistical Analysis

Statistical analysis was performed using SAS 9.4 (SAS Institute Inc., Cary, NC, USA). First, a descriptive analysis was performed to show frequency distributions and averages. Subsequently, the dependent variables of walking speed (WS) as well as the standard deviation in volume in the recorded footfall sound (SDV) were tested for normal distribution using histograms and a Shapiro-Wilk test. Afterwards, WS and SDV were analyzed with a general linear mixed model (PROC GLIMMIX) considering the potential influencing factors: group of housing (1 vs. 2), parity number (1–6), bodyweight, locomotion score (LS1, LS2, LS3) and type of disease (0–2). Multiple comparisons of least square means (LSMEANS) were performed using the post-hoc Tukey test. The results of the statistical tests were considered to be significant at *p*-values < 0.05.

## 3. Results

Installing the test track outside the stable offered the advantage of having a straight alley for observation and recording. However, this arrangement also resulted in an increased agility of the cows, even though they had been acclimatized to the test track in advance. Data were generated from 144 animals. Data of cows stopping, running, jumping or walking as a group on the measurement zone had to be deleted as this resulted in errors. Therefore, the walking sound of 76 animals (group 1: n = 36 and group 2: n = 40) was analyzed. These cows reached an average parity of 3 (min = 1; max = 6) and an average bodyweight of 685 kg (min = 538 kg; max = 880 kg). The distribution of cows affected by the different diseases within the rated locomotion scores and the parity numbers are shown in Table 1 and Table 2.

### 3.1. Walking Speed

**Figure 3:**
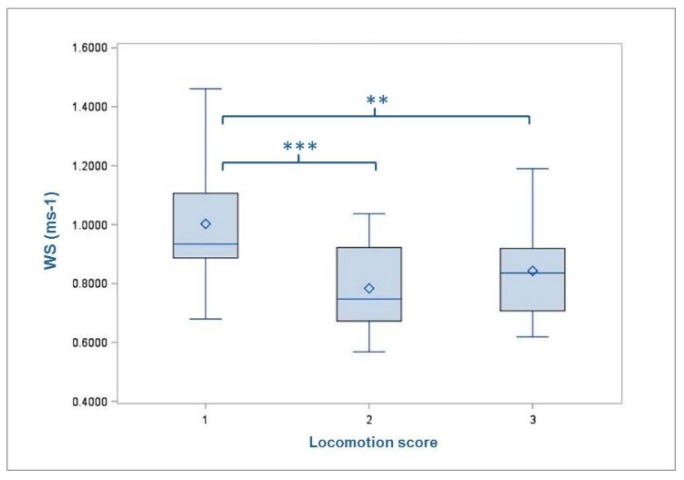
Mean walking speed (WS) of the cows with regard to the degree of rated locomotion score. Level of statistical significance between the LSMEANS is indicated by the number of asterisks (** *p* < 0.01; *** *p* < 0.001). The mean value of each LS-group is indicated by the following symbol: ◊ inside the boxes.

**Figure 4:**
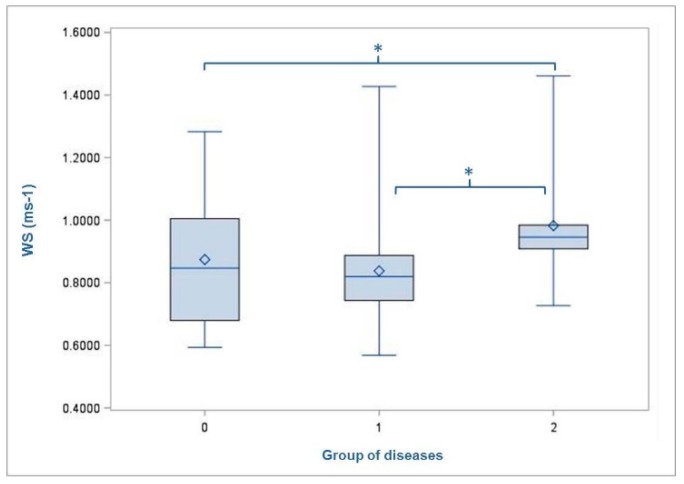
Mean walking speed (WS) of the cows with regard to the diagnosed type of diseases (group 0 = no diseases; group 1 = non-infectious diseases; group 2 = infectious diseases). Level of statistical significance between the LSMEANS is indicated by the number of asterisks (* *p* < 0.05). The mean value of each group of disease is indicated by the following symbol: ◊ inside the boxes.

The average time for passing the measuring section was 3.68 s (min = 2.12 s; max = 5.46 s; SD = 0.75). Accordingly, the cows covered the distance of the test track of 3.1 m at a speed of 0.84 ms^−1^. Thereby, the animals in group 1 (slatted floor; 0.85 ms^−1^) walked faster than those in group 2 (deep straw; 0.83 ms^−1^). Furthermore, the walking speed decreased with increasing parity number up to parity 5 (1 = 0.89 ms^−1^; 2 = 0.86 ms^−1^; 3 = 0.85 ms^−1^; 4 = 0.76 ms^−1^; 5 = 0.67 ms^−1^; 6 = 0.84 ms^−1^). The mean WS was significantly affected by the rated locomotion score (*p* = 0.0001) and the type of claw lesion (*p* = 0.0466) (Table 3). The comparisons of LSMEANS showed differences between LS1 (0.96 ms^−1^) and LS2 (0.76 ms^−1^) (*p* = < 0.0001) as well as between LS1 (0.96 ms^−1^) and LS3 (0.82 ms^−1^) (*p* = 0.0062), whereby the cows rated with LS1 were the fastest ones (Figure 3). Likewise, cows affected by non-infectious diseases (0.81 ms^−1^) showed a slower walk than those without diseases (0.83 ms^−1^) (p = 0.5649) and those with infectious disease (0.9 6 ms^−1^) (*p* = 0.0146) (Figure 4).

### 3.2. Standard Deviation of Volume of the Recorded Footfall Sound

**Figure 5:**
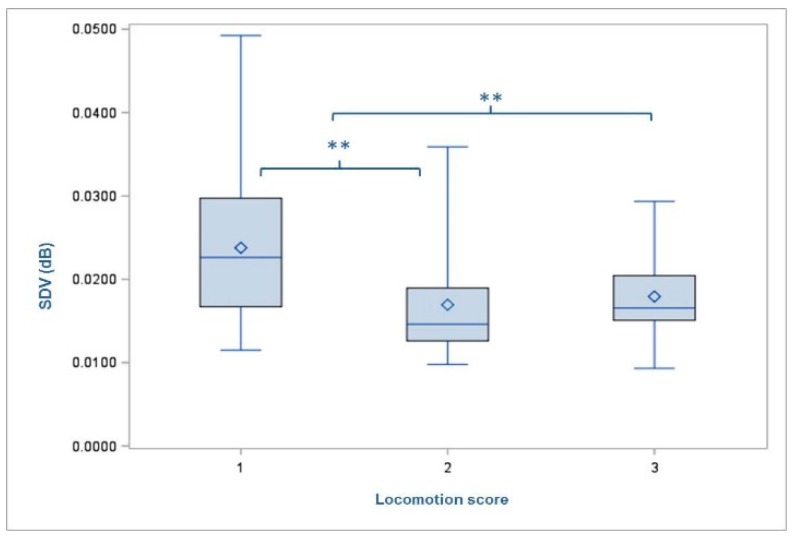
Mean standard deviation of volume (SDV) of the cows with regard to the degree of rated locomotion score. Level of statistical significance between the LSMEANS is indicated by the number of asterisks (** *p* < 0.001). The mean value of each locomotion score is indicated by the following symbol: ◊ inside the boxes.

**Figure 6:**
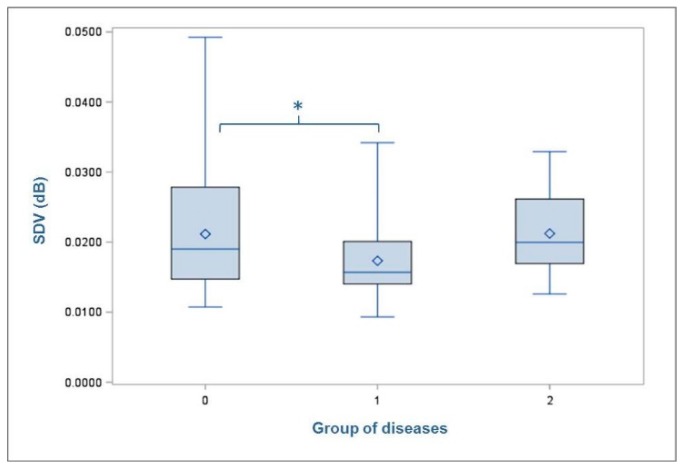
Mean standard deviation of volume (SDV) of the cows with regard to their claw lesions (group 0 = no diseases; group 1 = non-infectious diseases; group 2 = infectious diseases). Level of statistical significance between the LSMEANS is indicated by the number of asterisks (* *p* < 0.05). The mean value of each group of disease is indicated by the following symbol: ◊ inside the boxes.

The mean standard deviation in the recorded volume of footfall sound (SDV) was 0.019 dB (min = 0.009 dB; max = 0.049 dB; SD = 0.007). No differences in the cows’ footfall sound were found between the two housing groups. However, animals with increased parity number showed decreased SDV (1 = 0.020 dB; 2 = 0.020 dB; 3 = 0.019dB; 4 = 0.018 dB; 5 = 0.013 dB; 6 = 0.017 dB). The SDV was affected by the locomotion score (*p* = 0.0014) just like the WS. The comparisons of LSMEANS showed significant differences between LS1 (0.024 dB) and LS2 (0.017 dB) (*p* = 0.0032) as well as between LS1 (0.024 dB) and LS3 (0.018 dB) (*p* = 0.0060) with animals classified to the non-lame group (LS1) showing the loudest footfall (Figure 5). Furthermore, a trend towards an influence of the variable ‘types of diseases’ was detected with *p* = 0.0721. The comparisons of LSMEANS revealed that animals with non-infectious diseases (Group 1 = 0.017 dB) showed significantly lower SDV (*p* = 0.0366) than cows without any disease (Group 0 = 0.021 dB) (Figure 6). The other results (F- and *p*-values) of the test for significant differences are shown in Table 3.

## 4. Discussion

This approach showed that the recording of the footfall sound of cows with the installed system was possible. Each cow left an individual gait pattern on the solid surface which could be measured and examined. It was possible to decouple the recording of possible acoustic disturbances from any disturbing noises from the environment. Therefore, we found variations in the walking speed as well as differences in the maximum volume representing the weight loaded on each leg to differentiate non-lame and lame cows. Nonetheless, we also obtained suggestions for improvement through this first approach. For instance, the installation of the test track outside the stable proved not to be a practical solution for an undisturbed experimental procedure. The walk in this unfamiliar environment required the cows to become acclimatized to the test track. Despite the previous training, walking over the test track did not become completely normal for the cows as the error rate in the data showed. Perhaps the previous targeted habituation was not sufficiently long enough although in other studies two days was taken for this adaptation phase [17]. In contrast, Pastell et al. 2010 conducted trial runs for at least four days with four passages per day to acclimatize the animals to the procedure of their study [7]. However, for a practical experimental procedure it is advisable to place the test track in the cows’ familiar environment. Other studies propose using the way back from the milking parlor back to the stable or to pasture if it offers a straight plan section [9,18,19,20].

### 4.1. Walking Speed

In the present study, the average walking speed at the measurement zone was 0.84 ms^−1^ on a slatted concrete floor. This result corresponds to the speed on the concrete floor, measured in the morning, in the study of Zillner et al. (2018) [18]. On a concrete or slatted floor the claws have less contact to the ground surface as they cannot sink into the ground. Thus, the cows tend to show a slower respectively more careful walking speed than for example on a rubber surface [17]. Nevertheless, the cows in the present study generally walked slower than for example the animals on a slatted or concrete floor in the study by Telezhenko and Bergsten (2005) who compared the speed on different floor types [19]. A study by Chapinal et al. (2010) determined a walking speed of 1.42 ms^−1^ in non-lame cows [9]. These even high differences in the walking speed may result from the varying measurement methods. In our study the recordings started with the first step taken on the floor. In contrast, Chapinal et al. (2010) started the recording when the cows’ nose was seen at the starting mark on the video [9]. Another reason for the slower movement of the cows in the present study could be related to the already mentioned fact that the animals had to pass the test track outside the stable and, thus, in an unfamiliar environment. Furthermore, there was no human escort walking behind the cows to encourage them when necessary to move down the test track. A further explanation for the reduced speed in the present study can be found in the low motivation of the cows to return to the stable after milking to start with feed and water intake. In this study, the cows had to walk along the test track in the morning two hours after the first milking in the morning (of the cows from housing group 1 which were milked in the milking parlor). 

The comparisons of the walking speed of cows within the different housing types showed that the cows in group 1 (slatted floor) moved faster than those in the deep straw barn (group 2). One explanation may be that these cows had experience in walking over a slatted floor and did not walk as carefully as those in group 2. Moreover, animals with a higher parity number tended to have a decreased walking speed. Besides the decrease in agility of older cows, their increased udder sizes might force the animals to describe a circle with their hind legs to circumvent the udder [21,22]. This movement results in shorter steps, decreased movement ability and a reduced walking speed. Van Nuffel et al. (2016) also reported that older cows walked slower [22]. They stated that the age is definitely a cow-related factor which affects cows’ locomotion and not an environmental one [22]. 

In the present study, further differences in the walking speed were found between animals from different groups of claw diseases. Animals without claw lesions and cows affected by infectious diseases showed a faster movement than those affected by non-infectious disease such as sole ulcers. Accordingly, Flower et al. (2005) measured shorter strides and reduced speed of cows with sole ulcers compared to healthy animals [23]. Likewise, gait alterations in general, changes in walking speed included, were more evident in cows with sole ulcers than in animals without any lesion [24].

Finally, the rated locomotion score showed a significant influence on the WS. Cows assessed with LS1 passed the test track in significantly less time than animals rated with LS2 or LS3. This result particularly verifies the locomotion score because animals with a lower score represented a smoother and faster gait pattern. In other words: cows with a straight gait were rated better by the trained observer. These findings corresponded to results by Chapinal et al. (2009) [25]. They reported that walking speed is negatively correlated with the numerical gait score. Moreover, they considered that it is difficult to state whether gait changes are a consequence of slow gait or impaired gait results at a slow pace. In another study it was found that walking speed decreased during the experiments and, therefore, caution is advised when using this factor solely for lameness detection [9].

### 4.2. Standard Deviation of Volume of the Recorded Footfall Sound

The essential variable analyzed from the recorded footfall sound was the SDV which was considered as a factor for the force of a cow’s single footsteps. The higher this value was the louder the difference was between a sound signal and no sound signal. In the present study, cows with increased parity number showed decreased SDV. The older the animals were the less vigorous were their steps. This evidence corresponds to previous findings regarding the pedobarometric forces of cattle to the ground surface [26]. Distl and Mair (1993) measured that the maximum pressure in claws of the front right foot was higher in first lactating cows (58.6 N/cm³) than in second lactating cows (56.4 N/cm³), although the average force was lower in the younger animals [26]. However, the findings of our study become clearer when considering the prevalence of (non-infectious) diseases in each parity group. In general, there was an increasing tendency towards non-infectious diseases with increasing parity number. Additionally, within the group of the fifth lactation one animal was affected by sole ulcers with a high severity level on both hind legs. This cow showed a very low SDV value of 0.0127 dB. Similarly, previous studies found that older cows are generally at higher risk of lameness [20,27,28,29]. Espejo et al. (2006) even observed that prevalence of lameness increased on average at a rate of eight percent per lactation [20]. 

Moreover, our findings showed significant differences between the documented averaged SDV within the three levels of rated locomotion score. With increasing locomotion score the recorded SDV decreased. Accordingly, Maertens et al. (2011) found that all kinematic variables differed between the given gait scores [10]. These kinematic variables from their study also described nothing but cows’ locomotion by measuring for example stride length, abduction, step overlap or asymmetry. Hence, it is quite explicable that the average SDV in our study was different between the levels of locomotion score. Furthermore, the prevalence of non-infectious disease within these groups increased with a higher score. 

In addition, a relationship was found between the presence of non-infectious claw disease and a decreasing SDV. Although, other authors analyzed the effects of individual diseases [25,30], we classified the claw lesions according to infectious and non-infectious diseases as this division has been described in previous studies [2,29] as well as in the used keycode [16]. Our results showed that cows affected by non-infectious claw lesions reached significantly lower SDV than those without claw lesions. Our findings support previous results showing the effect of claw lesions and how cows distribute their weight on the affected respectively healthy leg [30,31]. Pastell et al. (2010) investigated the distribution of weight applied to a pair of legs when the cow was standing [7]. They found a negative correlation between the locomotion score and the weight loaded on a pair of legs. For cows with sole ulcers there even was a linear relation between these two characteristics. Their results showed that the analyzed variable of weight distribution could certainly be used to distinguish cows with no claw lesions from those with relatively severe sole ulcers. Blackie et al. (2013) observed that stride length was significantly shorter in cows with sole ulcers compared to those with no lesions or any other claw lesion, and affected cows had significantly higher locomotion scores as well [24]. Likewise, our results support the statement of Passos et al. (2017) that especially non-infectious claw diseases such as sole ulcers have clear impacts on the strain on limbs. Lame cows have a greater sensitivity to pain, in particular those with non-infectious claw diseases, [29] and demonstrate a less forceful gait pattern. Furthermore, the findings from the presented study confirmed that lame cows adopt the best compensatory movements to minimize pain and, therefore, show less variation in their movement [24] as shown in the decreased SDV. In summary, with SDV in the recorded signal of footfall sound interpreted as a variable for the loudness or the weight load of cows’ footstep these first results clearly show the potential of footfall sound analysis for lameness detection.

However, the performance of the system has to be validated with more cows and on other farms where the system could be installed inside the stable (e.g., on the way back from the milking parlor). Further work is required to assess the footfall sound of cows concerning also the number and uniformity of their steps to improve analyzing the recorded signals. Moreover, it should be noted that for this first practical approach only one data set was examined using the method of mean comparison. To improve the evaluation of this method in terms of lameness detection, more parameters should be considered.

## 5. Conclusions

Lameness is a serious issue in dairy cows and it still poses a problem in dairy production and in particular in animal welfare. Measuring and respectively detecting the percentages of lame cows on a farm could help farmers prevent lameness from increasing without being noticed. As visual monitoring of locomotion on farm is time-consuming and thus cost inefficient, the aim of the presented study was to develop a system in practice which is capable of detecting claw lesions in dairy cows by analyzing their footfall sound. This current investigation shows that the used measurement installation can record cows’ individual footfall sound without any noise interference. The analyses of the recorded sounds can be used to successfully distinguish between cows without claw lesions and those animals affected by non-infectious disease that are considered to be particular painful ones. Further research is required on this system to improve the measurements on farms and especially a more detailed interpretation of the recorded footfall sounds.

## Figures and Tables

**Table 1:** Distribution of the parity for the number of cows classified regarding the diagnosed type of claw lesions (group 0 = no diseases; group 1 = non-infectious diseases; group 2 = infectious diseases).

Variable	Sample Number	Group 0		Group 1		Group 2	
Parity	n	n	%	n	%	n	%
1	15	10	66.7	0	0	5	33.3
2	30	11	36.7	15	50.0	4	13.3
3	16	2	12.5	10	62.5	4	25.0
4	9	3	33.3	6	66.7	0	0
5	3	2	66.7	1	33.3	0	0
6	3	2	66.7	1	33.3	0	0

**Table 2:** Distribution of the rated locomotion scores (LS) for the number of cows classified with regard to the diagnosed type of claw lesions (group 0 = no diseases; group 1 = non-infectious diseases; group 2 = infectious diseases).

	Sample Number	Group 0		Group 1		Group 2	
LS	n	n	%	n	%	n	%
1	24	17	70.8	3	12.5	4	16.7
2	21	7	33.3	10	47.7	4	19.0
3	31	6	19.4	20	64.5	5	16.1

**Table 3:** Results (F- and *p*-values) of the test for significant differences between the means of walking speed on the measurement zone (WS) and the standard deviation of volume in the recorded sound signal (SVD).

	WS		SDV	
Variable	F-value	*p*-value	F-value	*p*-value
Group of housing	0.08	0.7834	0.01	0.9240
Parity number	1.94	0.0988	0.61	0.6933
Bodyweight	1.46	0.2410	0.92	0.6089
Locomotion score	10.31	0.0001	7.17	0.0014
Type of disease	3.20	0.0466	2.39	0.0759

## Supplementary Materials

The following are available online at https://www.mdpi.com/2076-2615/9/3/78/s1, Figure S1: Test track of slatted floor elements on buffers. Figure S2: Test track outside the stable showing the weighing platform in the front and the measurement zone at the middle element of slatted floor.
