# Supplementary figures and images for: Using the Footfall Sound of Dairy Cows for Detecting Claw Lesions
Source: Animals (Basel). 2019 Mar 1;9(3):78. doi: 10.3390/ani9030078 (PMC6466211; doi:10.3390/ani9030078)

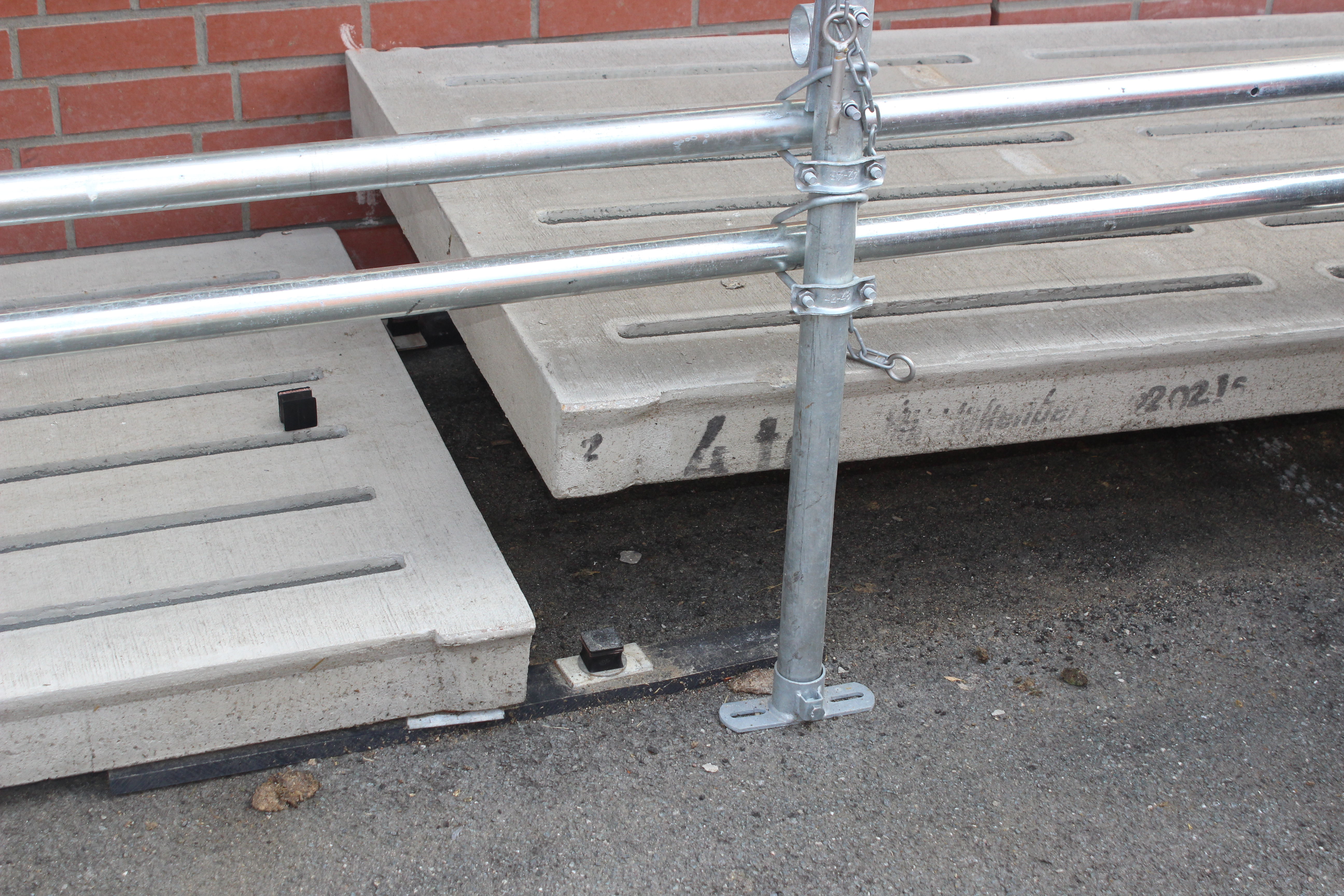

Supplement: Supplementary file 1 [file animals-09-00078-s001.zip › Figure_S1.jpg]

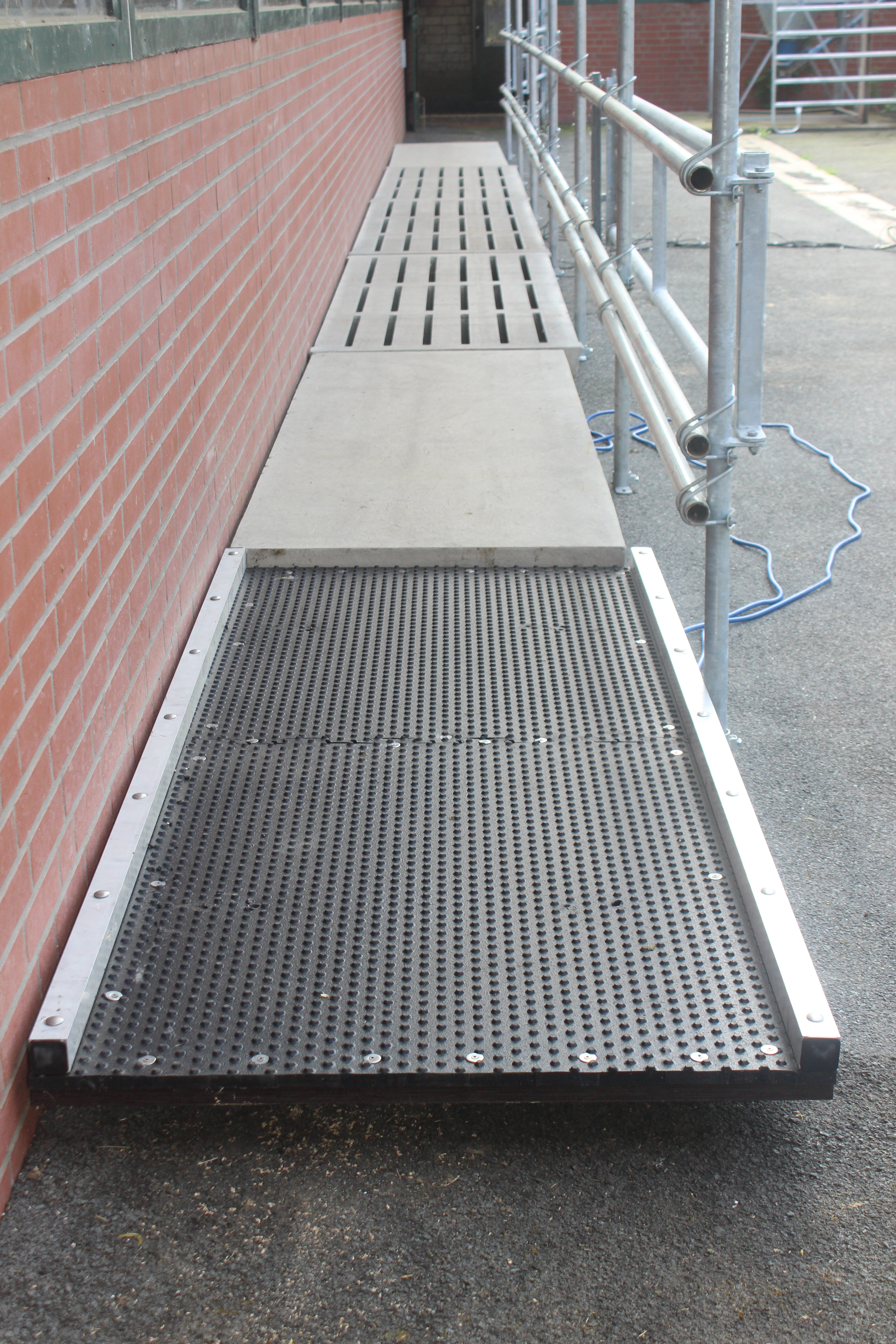

Supplement: Supplementary file 1 [file animals-09-00078-s001.zip › Figure_S2.jpg]
